# The Effect of Combination Therapy with Rituximab and Intravenous Immunoglobulin on the Progression of Chronic Antibody Mediated Rejection in Renal Transplant Recipients

**DOI:** 10.1155/2014/828732

**Published:** 2014-01-29

**Authors:** Gun Hee An, Jintak Yun, Yu Ah Hong, Marina Khvan, Byung Ha Chung, Bum Soon Choi, Cheol Whee Park, Yeong Jin Choi, Yong-Soo Kim, Chul Woo Yang

**Affiliations:** ^1^Division of Nephrology, Department of Internal Medicine, Seoul St. Mary's Hospital, College of Medicine, The Catholic University of Korea, Seoul, Republic of Korea; ^2^Division of Nephrology, Department of Internal Medicine, Korea University Guro Hospital, Seoul, Republic of Korea; ^3^Dialysis Department, National Research Center for Maternal and Child Health, Astana, Kazakhstan; ^4^Transplant Research Center, Seoul St. Mary's Hospital, College of Medicine, The Catholic University of Korea, Seoul, Republic of Korea; ^5^Department of Hospital Pathology, Seoul St. Mary's Hospital, College of Medicine, The Catholic University of Korea, Seoul, Republic of Korea

## Abstract

The treatment for chronic active antibody-mediated rejection (CAMR) remains controversial. We investigated the efficacy of rituximab (RTX) and intravenous immunoglobulin (IVIg) for CAMR. Eighteen patients with CAMR were treated with RTX (375 mg/m^2^) and IVIg (0.4 g/kg) for 4 days. The efficacy of RTX/IVIg combination therapy (RIT) was assessed by decline in estimated glomerular filtration rate per month (ΔeGFR) before and after RIT. Patients were divided into responder and nonresponder groups based on decrease and no decrease in ΔeGFR, respectively, and their clinical and histological characteristics were compared. Response rate to RIT was 66.7% (12/18), and overall ΔeGFR decreased significantly to 0.4 ± 1.7 mL*·*min^−1^ *·*1.73 m^−2^ per month 6 months after RIT compared to that observed 6 months before RIT (1.8 ± 1.0, *P* < 0.05). Clinical and histological features between the 12 responders and the 6 nonresponders were not significantly different, but nonresponders had a significantly higher proteinuria levels at the time of RIT (2.5 ± 2.5 versus 7.0 ± 3.5 protein/creatinine (g/g), *P* < 0.001). The effect of the RIT on ΔeGFR had dissipated in all patients by 1 year post-RIT. Thus, RIT delayed CAMR progression, and baseline proteinuria level was a prognostic factor for response to RIT.

## 1. Introduction

Circulating alloantibodies are found in a substantial number of renal allograft recipients, and the presence of these alloantibodies is significantly correlated with the development of allograft injury and later graft loss [1–3]. In renal allograft tissue, chronic injury is represented microscopically as transplant glomerulopathy and diffuse C4d deposition in peritubular capillaries (PTCs); recently, it was included as new disease entity named chronic antibody-mediated rejection (CAMR) in the update of the Banff 05 classification [4]. Usually the prognosis of CAMR is poor, and conventional immunosuppressants mainly targeting T cell-mediated immunity cannot prevent or reverse it [5–7]. Therefore, some researchers have suggested that therapies directed at the humoral response may be required for the treatment of CAMR [3].

Recently, some reports have suggested that the combined use of rituximab (RTX) and intravenous immunoglobulin (IVIg) therapy may be useful for the treatment of CAMR. Billing et al. published their experience with the RTX and IVIg combination protocol for treatment of CAMR in 6 pediatric patients, and they subsequently reported the long-term effects of this protocol [8, 9]. In adult renal transplant recipients, only a few studies have been published. Fehr et al. demonstrated that allograft function of CAMR was improved or stabilized with the RTX and IVIg combination therapy in 4 cases [10]. Our preliminary study also showed that the combination therapy was effective in delaying the progression of CAMR, especially in its early stages [11]. However, the above studies were conducted with small numbers of adult patients during periods of relatively short duration.

For these reasons, we decided to perform a study investigating the efficacy of the RTX and IVIg protocol for the treatment of CAMR, using a larger group of adult patients and with a longer period of followup.

## 2. Patients and Method

### 2.1. Diagnosis of CAMR

The diagnosis of CAMR was based on the update on Banff classification: (1) transplant glomerulopathy and severe peritubular capillary basement membrane multilayering (PTCBMM), interstitial fibrosis (IF) and tubular atrophy (TA) with or without peritubular capillary loss, and fibrous intimal thickening in arteries without internal elastic duplication; (2) diffuse C4d deposition in PTCs; and (3) presence of donor-specific anti-HLA antibody (DSA) [4]. Among allograft biopsies done between September 2009 and December 2012, in Seoul St. Mary's Hospital, 16 cases met the above Banff criteria. We also included 2 patients who did not fully satisfy with the criteria (negative HLA-DSA and C4d score 0 and score 1) but showed typical transplant glomerulopathy with slowly deteriorating graft function. Finally 18 patients were included in this study.

### 2.2. Patient Characteristics

Patient characteristics are shown in Table 1. The mean age of the patients was 44.0 ± 7.1 years at the time of CAMR diagnosis; 13 patients (72%) were male. Of the 18 patients, 11 (61%) received kidneys from living donors and 2 patients had histories of retransplantation. Eight of the 18 patients (44%) experienced acute rejection, including both antibody-mediated and T cell-mediated rejections, before CAMR. The median time posttransplant until the diagnosis of CAMR by renal graft biopsy was 93.2 months (range: 8.2–214.9). The follow-up duration after treatment was 14.1 months (range: 1.4–31.9). This study was approved by the Institutional Review Board of our institution (KC12RISI0070).

### 2.3. Protocol of Rituximab/IVIg Combination Therapy for CAMR

The protocol in our institution for the treatment of CAMR has been described previously (RIT protocol) [11]. Briefly, all patients were treated with IV RTX (375 mg/m^2^) once on day 1 followed by IVIg, 0.4 g/kg, once daily for 4 days. Pulse methylprednisolone at a dose of 500 mg IV was administered daily for the first 3 days, followed by oral prednisolone, tapered to 30 mg/day. We measured anti-HLA antibody using Luminex solid-phase assays (LSA; Tepnel Lifecodes Corp., Stamford, CT) at the time of biopsy. If the type of anti-HLA antibody detected in the patient corresponded to the HLA type of the donor, it was regarded as a donor-specific anti-HLA antibody (HLA-DSA). The results were presented as 4 levels, according to the median fluorescent intensity (MFI) value: strong, >10,000; moderate, 5000–10,000; weak, 1000–5000; and negative, <1000.

### 2.4. Efficacy of Treatment Protocol

The primary outcome of this study was improvement in allograft function after treatment. Allograft function was assessed on the basis of serum creatinine levels and estimated glomerular filtration rate (eGFR), using the modification of the diet in renal disease (MDRD) formula (eGFR = 186.3 ×  serum  creatinine^−1.154^×age^−0.263^ [×0.742 if female] mL·min^−1^·1.73 m^−2^) [12]. We calculated the decline in the rate of eGFR per month (ΔeGFR) during the 6 months before and after RIT and at 6-month intervals until the last followup. We also evaluated the amount of proteinuria (g protein/g creatinine (g/g)) in random urine chemistry, collected 6 months before RIT, at the time of RIT, 6 months after 6 RIT, and at 6-month intervals thereafter. Finally, we divided the patient populations into 2 groups, according to their response to the therapy. The responder group was comprised patients who showed a decrease of ΔeGFR during the 6 months after RIT; the patients who showed an increase or no decrease of ΔeGFR after treatment were regarded as nonresponder group. To investigate the factors associated with the response to the therapy, we compared (1) the clinical characteristics, (2) the change in allograft function and amount of proteinuria, (3) histopathologic findings, and (4) alloantibodies between the two groups.

### 2.5. Statistical Analysis

Data were expressed as mean and standard deviation (SD). Means of normally distributed data were compared using Student's *t*-test; a *χ* ^2^-test was used to compare proportions. The changes in eGFR before and after treatment were evaluated by paired comparison. Graft survival rates after RIT were calculated using Kaplan-Meier analysis, and we used the log-rank analysis to compare survival rates between groups. The association of the degree of histological lesions with the response to RIT was explored with Fisher's exact test. In all analyses, *P* < 0.05 (two-tailed) was taken to indicate statistical significance.

## 3. Results

### 3.1. Histologic and Immunologic Characteristics

Table 2 shows the histological characteristics at diagnosis of CAMR. Transplant glomerulopathy and PTCBMM were found in 10 patients (59%). Advanced chronic changes such as interstitial fibrosis and tubular atrophy were detected in most patients (17/18, 94%), and the staining for C4d was diffusely positive in 14 out of 18 patients (82%). In 16 patients who were examined for HLA-DSA using LSA at the time of biopsy, anti-HLA antibody was detected in 10 patients, and of these, 6 patients were identified to have HLA-DSA. HLA-DSA showed strong MFI in only 1 patient, moderate intensity in 3, and weak intensity in 2.

### 3.2. The Response to RTX/IVIG Treatment in Terms of Allograft Function

All patients tolerated RIT well and completed treatment without immediate adverse effects. Before RIT, progressive deterioration of allograft function was found in all patients. At 6 months before RIT, eGFR was 48.1 ± 17.5 mL·min^−1^·1.73 m^−2^ and progressively declined to 37.1 ± 15.6 mL·min^−1^·1.73 m^−2^ at the time of RIT (*P* < 0.001). The calculated ΔeGFR was 1.8 ± 1.0 mL·min^−1^·1.73 m^−2^ per month during that period. Six months after RIT, eGFR was 34.7 ± 19.2 mL·min^−1^·1.73 m^−2^, which is similar to that at the time of RIT (*P* = 0.40), and ΔeGFR 6 months after RIT was 0.4 ± 1.7 mL·min^−1^·1.73 m^−2^ per month, which is significantly lower than that at 6 months before RIT (*P* < 0.05). Proteinuria increased significantly from 3.0 ± 3.7 g/g at 6 months before RIT to 4.3 ± 3.6 g/g at the time of RIT (*P* < 0.05). The amount of proteinuria showed a decreasing trend at 6 months since RIT (3.0 ± 2.2 g/g, versus that at the time of RIT, *P* = 0.129) compared to the value at the time of RIT; this trend was observed even at the last followup (2.9 ± 2.7 g/g, versus at the time of RIT, *P* = 0.136).

### 3.3. Comparison between Responder and Nonresponder Groups

**Figure 1:**
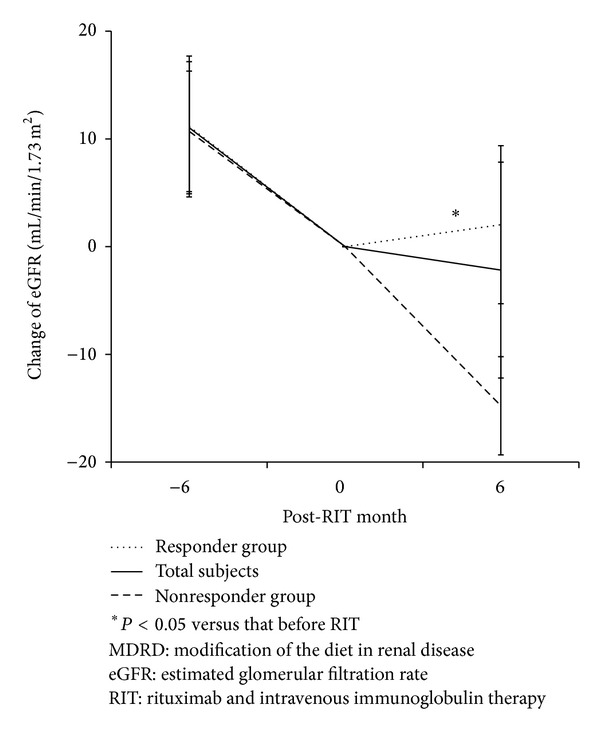
Comparison of changes in allograft function between the responder and nonresponder groups. eGFR showed sustained decline during 6 months before RIT in both responder and nonresponder groups. After RIT, eGFR in the responder group showed significant increase; however, decline of eGFR was persisted in the nonresponder group.

According to the change in ΔeGFR during 6 months after RIT compared to that observed 6 months before RIT, 12 patients (67%) met the criteria for the responder group, and the other 6 patients, for the nonresponder group. The eGFR at 6 months before RIT (45.4 ± 16.4 versus 54.5 ± 20.2 mL·min^−1^·1.73 m^−2^, *P* = 0.347) and that at the time of RIT (34.2 ± 14.3 versus 39.0 ± 20.3 mL·min^−1^·1.73 m^−2^, *P* = 0.568) were not significantly different between 2 groups. The ΔeGFR (1.9 ± 1.1 versus 1.8 ± 0.9 mL·min^−1^·1.73 m^−2^ per month, *P* = 0.83) 6 months before RIT did not differ between the 2 groups, as well. ΔeGFR decreased to −0.3 ± 1.2 mL·min^−1^·1.73 m^−2^ per month 6 months after RIT in the responder group compared to that observed 6 months before RIT (1.9 ± 1.1 mL·min^−1^·1.73 m^−2^ per month, *P* < 0.01). In contrast, nonresponders showed relatively higher ΔeGFRs 6 months after RIT (2.5 ± 0.8 mL·min^−1^·1.73 m^−2^ per month) compared to that before 6 months, which suggests that the allograft function was still rapidly deteriorating (*P* = 0.105; Figure 1). In comparison, the amount of proteinuria at the time of RIT was significantly higher in the nonresponder group (7.0 ± 3.5 g/g) than in the responder group (2.8 ± 2.8 g/g, *P* < 0.05). However, the histological features and other clinical parameters did not show any significant differences. The positivity of HLA-DSA at biopsy did not differ either (Table 3).

### 3.4. The Clinical Outcome during Long-Term Followup after Treatment

**Figure 2:**
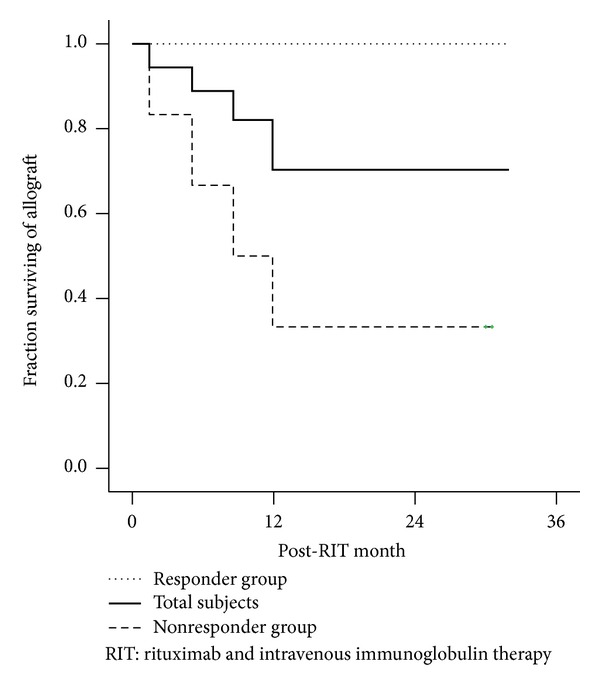
Allograft survival rate of the total number of patients and of those in the responder and nonresponder groups. During followup, the survival rate was 89%, 82%, and 70%, at 6, 12, and 24 months, respectively, in all patients. The responder group showed a significantly higher survival rate compared to the nonresponder group (*P* = 0.005).

**Figure 3:**
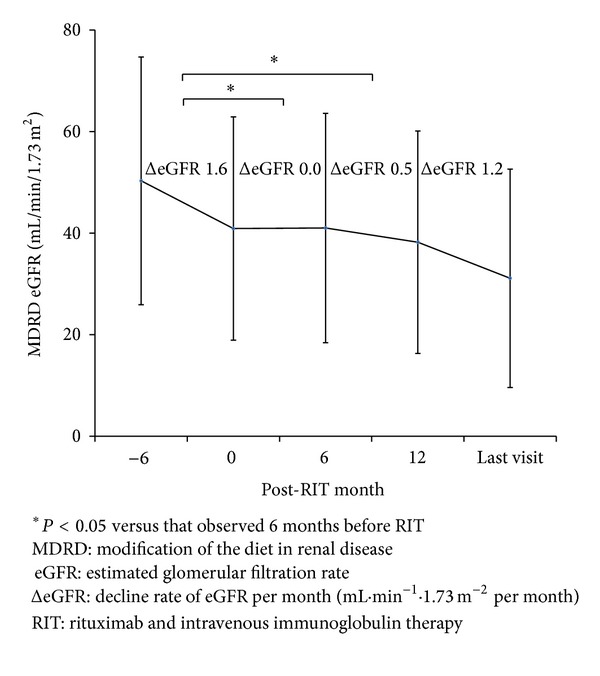
Changes in ΔeGFR values in 7 patients with long-term followup over 12 months. With time, ΔeGFR gradually decreased at every 6-month interval and the ΔeGFR at 12 months from RIT and that at the last followup showed similar values compared to that observed 6 months before RIT.

During long-term followup, only 1 case developed herpes zoster infection; no other serious complications were detected. Four patients (39%) exclusively in the nonresponder group experienced allograft loss at 1.4, 5.1, 8.6, and 11.9 months since treatment with RIT, and no allograft loss was noted in the responder group (Figure 2). In 7 patients with a follow-up duration of >12 months,the ΔeGFR observed 6–12 months after RIT (0.5 ± 0.7 mL·min^−1^·1.73 m^−2^ per month) was still lower than that observed 6 months before RIT (1.6 ± 1.1 mL·min^−1^·1.73 m^−2^ per month, *P* < 0.05). However, ΔeGFR showed an increasing trend over the final 12 months until the last followup (1.2 ± 0.8 mL·min^−1^·1.73 m^−2^ per month), at which it showed a value similar to that 6 months before RIT (Figure 3).

## 4. Discussion

In this study, 18 adult patients who were diagnosed as CAMR or suspicious of CAMR were treated with RTX and IVIg combination protocol. After this combination treatment, the rate of decline in allograft function decreased significantly in most patients, which suggests that this combination therapy is effective in delaying the progression of CAMR.

The effect of the combination therapy with RTX and IVIg on CAMR in pediatric patients has been reported in previous studies [8, 9]. However, the effect of the combination therapy in adult renal transplant recipients has not been established. We previously reported the beneficial effect of that therapy in 6 adult patients [11]. In this study, we investigated the effect of our protocol in larger patient group with longer follow-up period. The detailed mechanism for the development of CAMR has not been fully elucidated; however, in nature, antibody-mediated injury may be the main pathogenetic mechanism of CAMR [2, 3]. IVIg can suppress immunoglobulin synthesis, has anti-idiotypic activity against DSA (with resultant neutralization of DSA), blocks the Fc receptor, inhibits complement activation, and has anticytokine activity [13]. RTX, a chimeric anti-CD20 monoclonal antibody, can induce antibody-dependent cytotoxicity, complement-dependent cell killing, and apoptotic cell death, especially in B cells. Consequently RTX depletes B cells and interferes with antigen-presenting cell activity of B cells [14]. For this reason, RTX and IVIg, which target humoral immunity by different action mechanism, have been proposed as a therapeutic option for CAMR [8].

At first, we investigated the effect of this combination therapy on the progression of CAMR by comparing the rate of decline in eGFR before and after RIT. After RIT, the overall ΔeGFR slopped down, and in particular, in 12 out of 18 patients (67%), the ΔeGFR showed a significant decrease, which is similar to the result from a previous report [9]. The amount of proteinuria, which is poor prognostic factor for allograft outcome, showed a decrease after RIT, as well [15–17]. In addition, this protocol is well tolerated, and fatal infectious complications were not detected during the long-term follow-up period. All the above findings suggest that this protocol is not only effective but also safe for treating patients with CAMR.

However, 6 patients did not show a significant response to therapy and 4 out of the 6 patients in the nonresponder group experienced allograft failure within 1 year since treatment with RIT. To investigate the risk factors associated with this lack of response to the RIT protocol, we compared the clinical parameters between the responder and nonresponder groups. We did not find any significant differences in clinical characteristics. Of note, however, the amount of proteinuria at the time of RIT was significantly higher in nonresponder group than in the responder group. This finding is consistent with a previous study that showed that proteinuria is associated with more severe acute and chronic allograft rejection [18]. In contrast, allograft function at the time of RIT and the rate of decline in eGFR observed 6 months before RIT did not differ between the two groups. This suggests that the severity of allograft dysfunction does not predict the response to treatment.

In contrast to some previous reports, histological features were not associated with clinical outcomes in our study. For example, the proportion of transplant glomerulopathy and the severity of IF/TA, which is an important morphologic pattern of chronic kidney allograft injury, did not differ between the two groups [5, 19–21]. This result suggests that the histologic pattern is a prognostic factor in CAMR that progresses without intervention; however, it may not be an accurate prognostic indicator with the use of antihumoral therapy. Indeed, a previous study reported that pathological correlations that predicted the response to therapy were not identified [22]. Further investigation may be required to clarify this issue.

In the long-term followup, the therapeutic effect of RIT showed a decreasing trend with time, especially after 1 year since RIT initiation. In this study, 4 patients with a follow-up duration >2 years were included, and the time-dependent decrease in eGFR was detected. Interestingly, this pattern was found 6 months after RIT treatment not only in the nonresponder group but also in the responder group. The decrease may be associated with the duration of the B cell-depleting effect of RTX. A previous study showing RTX-induced B cell depletion in the peripheral blood indicated that patients recover approximately 6 months since RTX infusion [23], which suggests that the therapeutic effect of RIT on the progression of CAMR may be limited to this time period; accordingly, repeated RIT therapy or other additional strategies for humoral immunity such as bortezomib may be necessary to prolong the therapeutic effect [24–27].

The combination of RTX and IVIg showed a relatively long-term effect in pediatric renal transplant recipients with CAMR over 2 years, in contrast to this study [8, 9]. The possible reason is that the response to RIT may differ between adult and pediatric CAMR patients. The hematopoietic bone marrow contains mostly naive B cells of diverse specificities and has only a small number of memory B cell clones in childhood. Usually, memory B cells and plasma cell, which are responsible for the development of CAMR, accumulate with age [28]. Hence, these different immunologic characteristics, the higher memory B cell, and plasma cell pool in adult patients, may be associated with the limited long-term effect to RIT [29].

In our study, we included two patients who were not satisfied with the diagnostic criteria of CAMR; they did not show C4d deposition on biopsy tissue and DSA was not detected. We enrolled those patients for two reasons. First, it is strongly suggested that typical transplant glomerulopathy on allograft biopsy is responsible for slowly deteriorating allograft function. Second, there is a possibility that C4d or alloantibody is not detected even in the presence of morphologic evidence of antibody-mediated rejection [30]. It suggests that CAMR is a dynamic process and is difficult to make a clear-cut diagnosis.

This study has some limitations. First, we did not perform follow-up biopsies. Despite a significant decrease in ΔeGFR, we could not prove this benefit in the allograft tissue, for example, in the reduction of positive C4d or transplant glomerulopathy. Second, we did not include an untreated control group with CAMR. A larger randomized study, including treated subjects and untreated controls, may be required to prove the efficacy of RIT.

In conclusion, this study showed that the combination of RTX and IVIg is an effective treatment in delaying the progression of CAMR. In addition, the amount of proteinuria at the time of treatment is the most important prognostic factor for predicting the patient's response to RTX/IVIG combination therapy. However, the therapeutic effect showed a decreasing pattern over 1 year after RIT, which indicates that additional therapeutic strategy may be required in such patients.

## Figures and Tables

**Table 1:** Baseline characteristics of patients populations at treatment of CAMR.

Clinical parameters	All patients (*n* = 18)
Age (years)	44.0 ± 7.1
Male gender, *n* (%)	13 (72)
BMI (Kg/m^2^)	24.3 ± 2.7
Primary renal disease	
cGN, *n* (%)	7 (39)
HBP, *n* (%)	6 (33)
DM, *n* (%)	1 (6)
Unknown, *n* (%)	4 (22)
Dialysis type before KT	
Hemodialysis, *n* (%)	13 (72)
Peritoneal dialysis, *n* (%)	5 (28)
Dialysis duration, month	24.6 ± 24.5
Donor type, Living, *n* (%)	11 (61)
Multitransplant History, *n* (%)	2 (11)
Main immunosuppressant	
Cyclosporine, *n* (%)	6 (33)
Tacrolimus, *n* (%)	12 (67)
Previous acute rejection, *n* (%)	8 (44)
Serum Cr (mg/dL)	2.3 ± 0.9
MDRD eGFR (mL/min//1.73 m^2^)	35.8 ± 16.1
Proteinuria (g/day)	4.3 ± 3.6
Time posttransplant until diagnosis, month	93.2 ± 61.5
Time posttreatment, month	14.1 ± 9.3
HLA mismatch number	3.2 ± 1.4
HLA-DSA	
Not done, *n* (%)	2 (11)
Positive, Class I, *n* (%)	2 (11)
Positive, Class II, *n* (%)	5 (28)
Negative, *n* (%)	9 (50)

CAMR: chronic antibody mediate rejection; BMI: body mass index; cGN: chronic glomerulonephritis; Cr: creatinine; MDRD eGFR: estimated GFR using the Modification of Diet in Renal Disease Study equation; HLA-DSA: donor specific anti-HLA antibody.

**Table 2:** Histopathology of allograft biopsy and grading according to Banff 05′.

Characteristics (total *n* = 18)*	Score	*N* (%)
Transplant glomerulopathy (cg)	0	7 (41)
	1	0 (0)
	2	0 (0)
	3	10 (59)

PTC BMM	(−)	7 (41)
	(+)	10 (59)

C4d in PTC	0	3 (18)
	1	3 (18)
	2	9 (53)
	3	2 (11)

Peritubular capillaritis (ptc)	0	4 (24)
	1	1 (6)
	2	5 (29)
	3	7 (41)

Interstitial fibrosis (ci)/Tubular atrophy (ct)	0	1 (6)
	1	9 (50)
	2	7 (38)
	3	1 (6)

PTC: peritubular capillary; BMM: basement membrane multilayering.

*17 subjects had available data about transplant glomerulopathy, PTC BMM and C4d in PTC.

**Table 3:** Comparison of parameters between responder and nonresponder groups at treatment of CAMR.

Clinical parameters	Responder (*n* = 12)	Nonresponder (*n* = 6)	*P* value
Age (years)	44.0 ± 7.0	44.3 ± 7.9	0.928
Male gender, *n* (%)	8 (67)	5 (83)	0.615
BMI (Kg/m^2^)	24.0 ± 3.2	24.9 ± 1.8	0.518
Multitransplant History, *n* (%)	1.0 ± 0.0	1.3 ± 0.5	0.175
Previous acute rejection, *n* (%)	0.5 ± 0.7	0.7 ± 0.8	0.650
Serum Cr (mg/dL)	2.3 ± 0.7	2.4 ± 1.3	0.809
MDRD eGFR (mL/min//1.73 m^2^)	34.2 ± 14.3	39.0 ± 20.3	0.568
Proteinuira (g/day)	2.8 ± 2.8	7.0 ± 3.5	0.015
Time posttransplant before CAMR diagnosis, month	106.1 ± 65.6	67.3 ± 46.8	0.217
Time posttreatment, month	13.9 ± 7.8	14.6 ± 12.6	0.889

HLA-DSA, MFI*			0.629
Strong, *n* (%)	1 (10)	0 (0)	
Moderate, *n* (%)	1 (10)	2 (50)	
Weak, *n* (%)	2 (20)	0 (0)	
Negative, *n* (%)	6 (60)	2 (50)	

Histologic parameters			
Transplant glomerulopathy	1.75 ± 1.5	1.8 ± 1.6	0.953
PTCBMM (+/−)	7/5	3/2	0.951
Peritubular capillaritis	1.9 ± 1.3	1.8 ± 1.1	0.864
IF/TA	1.4 ± 0.8	1.5 ± 0.5	0.821
C4d in PTC	2.25 ± 1.4	2.8 ± 0.8	0.436

CAMR: chronic antibody mediate rejection; BMI: body mass index; Cr: creatinine; MDRD eGFR: estimated GFR using the Modification of Diet in Renal Disease Study equation; HLA-DSA: donor specific anti-HLA antibody; PTC: peritubular capillary; BMM: basement membrane multilayering.

*16 out of 18 subjects take HLA-DSA and 14 had available data.
